# Mapping antibiotic pollution and tracking drivers of environmental AMR in a North Indian pharmaceutical hub

**DOI:** 10.3389/fmicb.2025.1658029

**Published:** 2025-09-15

**Authors:** Amishi Panwar, Cansu Uluseker, Gian Singh Negi, Chiara Borsetto, Neelam Taneja, Helen Lambert

**Affiliations:** ^1^Department of Population Health Sciences, Bristol Medical School, University of Bristol, Bristol, England, United Kingdom; ^2^UK Centre for Ecology and Hydrology, Lancaster Environment Centre, Lancaster, England, United Kingdom; ^3^Department of Medical Microbiology, Post Graduate Institute of Medical Education and Research (PGIMER), Chandigarh, India; ^4^School of Life Sciences, Faculty of Science, Engineering and Medicine, University of Warwick, Warwick, England, United Kingdom; ^5^Department of Medical Microbiology, Post Graduate Institute of Medical Education and Research (PGIMER), Chandigarh, India; ^6^Department of Population Health Sciences, Bristol Medical School, University of Bristol, Bristol, England, United Kingdom

**Keywords:** antimicrobials, AMR, environment, antibiotic pollution, supply chains, India

## Abstract

**Introduction:**

Antibiotic residues in the environment contribute to the escalating threat of antimicrobial resistance (AMR), posing significant risks to public health and ecological systems. Understanding how antibiotics enter the environment during their lifecycle is crucial for developing effective mitigation strategies. This paper maps antibiotic pollution pathways in Baddi, Himachal Pradesh, highlighting the potential environmental and human health impacts of antimicrobial production, consumption, and disposal.

**Methods:**

A mixed-methods approach was used, combining qualitative interviews with stakeholders and community members, and quantitative analysis of sales data. Interviews with manufacturers, healthcare providers, and waste management officials provided insights into practices and perceptions related to antibiotic use and disposal. Sales data of four locally manufactured or packaged antibiotics during the COVID-19 pandemic were analyzed to trace their journey through the supply chain and identify potential points of environmental entry for antibiotic residues.

**Results:**

The study identified several critical points in the antibiotic supply chain where residues could enter the environment, including manufacturing discharge, disposal practices by consumers, and inadequate waste management systems. The analysis revealed an increase in antibiotic consumption during the COVID-19 pandemic, exacerbating the potential environmental burden. Key areas requiring oversight and management were highlighted, such as the need for better waste treatment facilities and stricter regulatory controls.

**Discussion:**

This research emphasizes the urgent need for a coordinated response at both the state and national levels to enhance environmental monitoring, improve waste management practices, and strengthen regulatory frameworks in India. Addressing these issues is essential to mitigate the growing threat of AMR and protect both environmental and human health. The findings advocate for comprehensive strategies involving multiple stakeholders to ensure sustainable management of antibiotics throughout their lifecycle.

## 1 Background

The existence of antibiotics in the environment raises alarm due to the potential emergence of antibiotic-resistant bacteria. With a diversity of huge bacterial populations and complex microbial and genetic interactions, the environment provides ideal conditions for gene development and exchange between indigenous microorganisms and those from humans and animals (Li and Zhang, 2023). These bacteria can proliferate in the environment and potentially transmit resistance genes to human pathogens, thereby complicating future treatments for bacterial infections. The risk encompasses the whole ecosystem, including humans, wildlife, and aquatic life. Antibiotic resistance is a type of Antimicrobial Resistance (AMR) specific to bacteria, and AMR disregards geographical borders and can disseminate worldwide, threatening global health security. The World Health Organization (WHO) has declared antimicrobial resistance (AMR) a critical global health threat, warning that it undermines progress toward Sustainable Development Goal (SDG) 3, which aims to “ensure healthy lives and promote wellbeing for all at all ages,” including the prevention of deaths and illnesses caused by environmental pollution and infectious diseases (World Health Organization, 2019). India and other countries in the South Asian region have reported high levels of antibiotic consumption, residues of which find their way into wastewater systems (Taneja and Sharma, 2019; Barathe et al., 2024). They could lead to the development of resistance genes in the environment, impacting the entire ecosystem, including human and animal health. Keeping in mind the complex supply chain and the One Health framework^1^ of addressing AMR, this research considers explicitly the role of the pharmaceutical industry's production of antibiotics in relation to its implications for the Sustainable Development Goals (SDGs). SDGs being addressed include Target 3.9 (Reduce the number of deaths and illnesses from hazardous chemicals and air, water and soil pollution and contamination) and SDG 12 (responsible consumption, production, and waste practices), SDG 8 (sustainable economic growth), and SDG 10 (reduced inequalities).^2^

The presence of antibiotics in the environment is particularly concerning because, even at sub-inhibitory concentrations, they can create selective pressure on microbial communities, favoring the survival and proliferation of antibiotic-resistant bacteria (ARB) and the dissemination of antibiotic resistance genes (ARGs; Martinez, 2009; Tello et al., 2012). Environmental compartments such as soil, rivers, sediments, and wastewater can act as reservoirs where resistance traits are exchanged among native microorganisms and bacteria originating from human and animal waste (Rizzo et al., 2013; Uluseker et al., 2021). Human and animal excreta also contribute ARB and ARGs to these environments, compounding the risk of resistance proliferation (Pruden et al., 2006). The coexistence of antibiotic residues with ARB and ARGs in environmental matrices accelerates the selection and persistence of resistant strains, which may subsequently transfer resistance to clinically relevant pathogens, posing a broader risk to human, animal, and ecosystem health (Kraemer et al., 2019). Poor wastewater management in India directly discharges untreated or inadequately treated sewage into rivers, lakes, and groundwater, resulting in antibiotic contamination. As a 2024 systematic review on *Mapping the scarcity of data on antibiotics in natural and engineered water environments across India* (Rathinavelu et al., 2024) shows, major river systems, including the Yamuna, Ganga, Musi, and others across North, Central, South, and West India, have been found to contain varying concentrations of antibiotics due to industrial, domestic, and hospital waste. Seasonal variations influence antibiotic presence, and while contamination in drinking water is below detection limits, antibiotics have been widely detected in lakes, ponds, tanks, and groundwater, posing environmental and health risks, emphasizing the need for proper disposal and regulation to prevent AMR and environmental damage (Rathinavelu et al., 2024).

The journey of antimicrobials into and through the environment is intricately linked with the multifaceted antibiotic supply chain (Wu-Wu et al., 2023; Zhang, 2023; Yang et al., 2022), from the creation of Active Pharmaceutical Ingredients (APIs) to the production, distribution, usage, and disposal of antimicrobial drugs. In India, this supply chain lacks thorough documentation, necessitating a detailed mapping to pinpoint where antibiotic production materials and remnants—commonly referred to as “waste”—may infiltrate the environment. Humans or animals do not entirely metabolize consumed antibiotics; a substantial fraction is expelled and finds its way into wastewater, where it cannot be entirely removed (Yang et al., 2021; Kümmerer, 2009). In India, inadequate waste management and high antibiotic use contribute significantly to environmental antibiotic pollution, exacerbating antimicrobial resistance (AMR; Taneja and Sharma, 2019; Barathe et al., 2024; Cameron et al., 2025). A large fraction of sewage is discharged untreated into water bodies, and existing wastewater treatment plants (STPs) are often ineffective in removing antibiotics.

Additionally, widespread over-the-counter antibiotic sales, self-medication, and extensive use in agriculture and animal husbandry further increase antibiotic contamination. Strengthening waste treatment infrastructure, enforcing zero liquid discharge in pharmaceutical industries, and promoting responsible antibiotic use are crucial to mitigating environmental AMR (Rathinavelu et al., 2024). In areas like Himachal Pradesh, particularly in industrial and pharmaceutical centers like Baddi, the environmental concentration of these pharmaceuticals can be exceptionally high and may contribute to AMR (Sinha, 2017). This is attributable to both local antibiotic production methods and the disposal practices of unused drugs.

Communities residing near pharmaceutical production sites may face a heightened risk of potential exposure to elevated levels of antibiotics and antibiotic-resistant bacteria in their surroundings. Comprehending the local trends of antibiotic retailing and dispensing from a “bottom-up” perspective aids in identifying potential risk points for the environmental discharge of antimicrobials. This paper aims to chronicle the sources and pathways of antibiotic formulation and production, the procurement of antibiotics by local health providers in the residential areas involved in our study, and their path to the point of utilization or disposal. This offers a crucial foundation for better understanding the scope of environmental antimicrobial pollution and elucidates where responsibilities for improvements in monitoring antibiotic residues, public health management, and environmental protection lie.

## 2 Materials and methods

**Figure 1:**
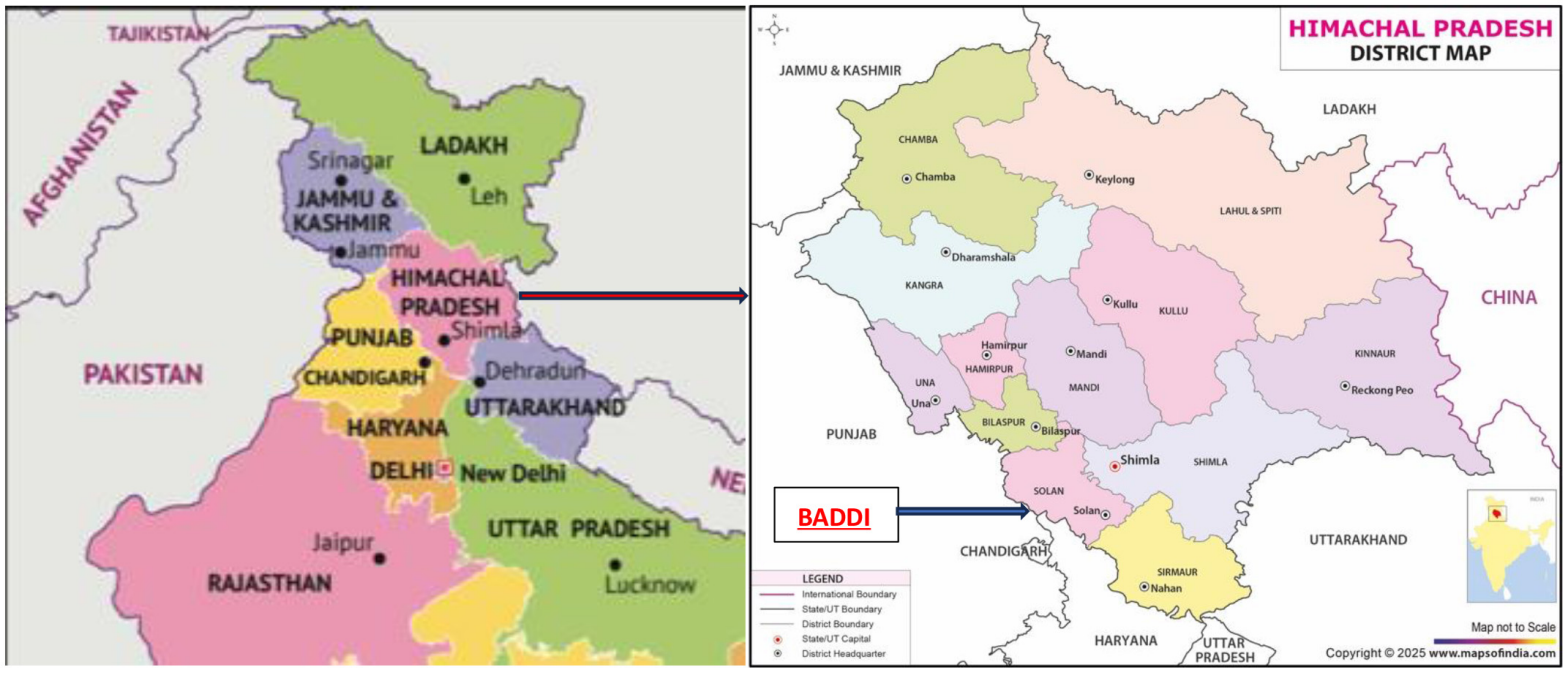
**(A)** Map of Northern India (Maps of India, 2025b), **(B)** District map of Himachal Pradesh (Maps of India, 2025a).

Baddi, one of Asia's biggest pharmaceutical hubs, is located near the Sirsa River about 260 km north of New Delhi (see Figure 1). As a pharmaceutical hub, companies in Baddi manufacture formulations, or finished products such as tablets, syrups, and topical medication. Only one company manufactures APIs, or the main biologically active ingredient used in formulations, including antibiotics.

**Figure 2:**
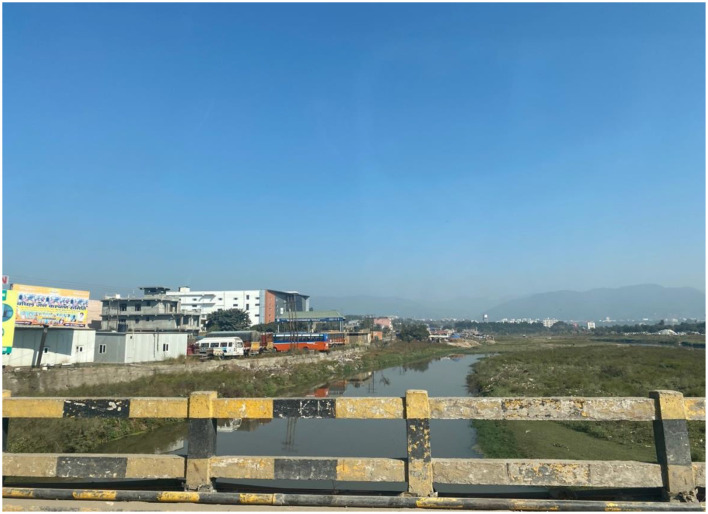
Entry to the Baddi pharmaceutical hub on the banks of the Sirsa river (photo by Amishi Panwar 2022).

This study is part of a larger programme of research^3^ funded by the Department of Biotechnology, Government of India, and the National Environment Research Council, United Kingdom, entitled “*Tackling AMR in the environment from antimicrobial manufacturing waste”* (2019–2023). Five bilateral projects were funded starting in 2020. This paper presents findings from a collaborative study between two projects, ResPHARM^4^ *(Resolving the fate and studying the impact of pharmaceutical wastes on the environment and a local community of a pharmaceutical manufacturing hub) and* AMRflows.^5^ AMRflows studied antimicrobials and resistance from manufacturing flows to people through risk analysis combined with field sampling, mesocosm, and laboratory studies with mathematical modeling to quantify the dynamics of resistant bacteria, plasmid transfer, and antibiotics in rivers impacted by pharmaceutical wastes. ResPharm aimed to document direct exposure to antibiotics through consumption and indirect exposure through ingestion (via food and drinking water) of antibiotic residues emitted from industrial processes or waste disposal into the environment. The field sites for comparison are Baddi^6^ and the unindustrialised town of Kangra in the Himalayan foothills of Himachal Pradesh. However, the comparative analysis of Baddi and Kangra is part of the wider project ResPharm and is not within the scope of this article (Figure 2).

Sales data (provided as part of project AMRflows) from Quintiles and IMS Health, Inc. (IQVIA) were obtained for the state and municipal areas of Himachal Pradesh and Baddi to document the wholesale distribution and consumption of antibiotics. The IQVIA database records the volume of antibiotic molecule sales utilized in human medicine based on pharmacist transactions. IQVIA data are collected from a network of stockists who sell pharmaceuticals to pharmacies, whether over the counter or based on prescriptions. The data include a list of antibiotics sold and their corresponding amounts for each month and year during the study period. The total antibiotic concentration is calculated by multiplying the number of sold packs by antibiotic units in each sold pack. Four commonly used antibiotics were identified for this study. Ciprofloxacin and Doxycycline were selected together with Amoxicillin and Azithromycin after a pilot study (qualitative) in the Baddi area.

Baddi, being a smaller industrial city, has significantly lower antibiotic consumption compared to larger urban centers (when compared with IQVIA sales data from other cities in India). Its sales figures are many times lower than those of major cities, reflecting differences in population size, healthcare infrastructure, and medical demand. For instance, Baddi's Amoxicillin, Azithromycin, and Ciprofloxacin sales are only a fraction of what is sold in bigger cities, often hundreds of times lower. Even the least-sold antibiotic, Doxycycline, shows a vast gap (see Figure 4), with Baddi consuming far less than its urban counterparts. This stark contrast highlights how larger cities drive high pharmaceutical demand due to their extensive healthcare networks and larger populations, whereas Baddi's usage remains minimal in comparison.

To analyze temporal trends in antibiotic sales and to forecast likely changes, linear trend estimations were performed. Monthly sales volumes (in antibiotic units) for each antibiotic were compiled for the study period. For each molecule, a simple linear regression model was applied to estimate the slope of sales over time, allowing for the visualization of increases or decreases in consumption. Forecasts were generated by extending the regression line to project sales trends beyond the observed data period. This approach provided a straightforward method to identify patterns and compare the growth or decline of antibiotic use across different molecules and locations.

The quantitative analysis of antibiotic sales trends provides a solid foundation for understanding the broad patterns of antimicrobial consumption, complemented by qualitative data from field sites in Baddi. Qualitative interviews and ethnographic observations of community level behaviors and practices behind these patterns, enable the construction of a more holistic view of the drivers of antibiotic consumption and disposal. Thirty interviews^7^ were conducted from March to August 2023 with stakeholders in Baddi and Chandigarh, and community members in field sites close to environmental sampling locations where high concentrations of antibiotic residues were found. At the start of project ResPharm, we comprehensively mapped the water catchment area in and around Baddi and identified relevant features including hospitals, pharmacies, municipal sewage treatment plants, factories etc. We then selected points for environmental (water and silt) sampling that we identified with GIS locators; these included sampling points where pharmaceutical and domestic waste was discharged into the river, up- and downstream of wastewater treatment plants and factories, etc. After the first round of sampling, we reduced the total number of sampling points to focus on locations where chemical analysis of these environmental samples had provided evidence of heavier pharmaceutical pollution. Site-specific observation and interviews then commenced at locations in the vicinity of these sampling points.

Given the restricted access to production sites in many pharmaceutical companies, this project aimed to understand the manufacturing and production of antibiotics through interviews with pharmaceutical company owners, pharmaceutical company managers, and antimicrobial manufacturers. Information about antimicrobial production was gathered by interviewing four antimicrobial manufacturers about the sources of constituent components and raw materials. In documenting ingredient procurement and manufacturing processes, we also identified “waste disposal” processes or emission points, and points of domestic disposal of medicines or antibiotics. More general data about production lines, packaging, onward distribution, and quality control measures were collected by interviewing three pharmaceutical company managers (Table 1).

**Table 1:** Summary of participants and methods used in the study.

**Study participants**	**Method**	**Number**
Antimicrobial manufacturers	In-depth interview	4
Pharmaceutical company managers	In-depth interview	3
Community doctors	In-depth interview	4
Pharmacists	In-depth interview	5
Slum dwellers	In-depth interview	6
Solid waste treatment plant heads	In-depth interview	2
Liquid waste treatment plant head	In-depth interview	1
Drug control officials	In-depth interview	3
Pollution control board officials	In-depth interview	2
**Total**		**30**

As was evident from initial conversations with pharmaceutical company managers, there was a rise in the production of specific antibiotics during and post-COVID-19, as the usage of amoxicillin and azithromycin was particularly high (self-medication for the treatment of COVID-19 and similar symptoms). This trend was matched with IQVIA sales data for the four antibiotics in Baddi and Himachal Pradesh, which helped us understand the percentage of sales since 2015. This was further substantiated with 15 interviews in community settings that included four local community doctors, five pharmacists, and six slum dwellers using any of the four antibiotics. Sanitation measures, as observed on the field, also provided an additional point of entry into the environment. Since manufacturing processes could not be directly observed, interviews with factory workers living in the residential communities that supply labor to this industry were conducted to gain additional insights. Information on local procurement, retailing, and prescribing of antibiotics was collected from pharmacies and local doctors in our Himachal Pradesh field sites. This process thus provided a map of the local supply chain wherein we methodically traced both the sale of selected antibiotics based on their consumption patterns and identified disposal practices through which antimicrobials may enter the environment.

For tracing the disposal of antibiotic effluent waste, three interviews were conducted with solid and liquid waste treatment plant heads to map the spread of antibiotic residues. Three interviews with drug controllers and two interviews with pollution control board officials were conducted to better understand quality control and the extent of environmental pollution due to pharmaceutical waste. Verbal consent was sought from stakeholders and community members, followed by a brief explanation of their role in this research, the right to withdraw at any point during the research, and the right to anonymity. All names are anonymized except in cases where permission was granted. The data were coded and analyzed thematically to identify health risks associated with environmental contamination, especially pharmaceutical waste.

## 3 Results

### 3.1 Antibiotic manufacturing, sales and environmental pollution processes

Overall, the Indian pharmaceutical industry is thought to supply 20% of generic drugs globally (Subramanian, 2023; Bjerke, 2022). Roughly 1,500 units across the country are involved in “bulk manufacturing” of antimicrobials—that is, the creation of the Active Pharmaceutical Ingredients (APIs) present in these drugs, which is likely to be the most hazardous process in terms of potential environmental pollution (James, 2020). An “Active Pharmaceutical Ingredient” (API) is a chemical molecule in the pharmaceutical product (TNN, 2002) while a “bulk drug” means “any substance that is represented for use in a drug and that, when used in the manufacturing, processing, or packaging of a drug, becomes an active ingredient or a finished dosage form of the drug” (Cornell Law School, 2014). A pharmaceutical company employee who had moved to Baddi for work stated,

*[...] Currently, our dependency on external sources (referring to raw materials) persists. We cannot rely solely on India for all types of salts (term often used interchangeably with APIs). Some salts, such as paracetamol, cannot be produced domestically, necessitating imports. The required quantities are not readily available within our resources, especially considering India's population of over 1.4 billion, which generates significant demand [...] The domestic supply falls short of meeting the demand, making it impractical for everyone to rely solely on these sources. High-level companies prefer to directly import from overseas as it provides easier access and quicker availability, not just due to cost considerations but also due to their substantial demand and time constraints. While we are not entirely dependent on India alone, we are still reliant on external sources, including China*.

The first step to bulk drug production is acquiring raw materials that are purified to produce substances like APIs. The availability of limited resources, like APIs and other raw materials for making antibiotics, was further put to the test during the COVID-19 pandemic and the years following it. The COVID-19 pandemic highlighted the need for India to enhance its sources of materials for pharmaceutical manufacturing, but consequent antibiotic pollution in the environment from manufacturing waste remained a concern.

We spoke to a scrap dealer based in Baddi, who deals in waste and waste material from the production of medicines and is also involved in the bulk production of Nimesulide powder, a commonly used ingredient in painkillers and anti-fever medication, classified under the red zone (activity that produces hazardous waste^8^) in India. According to this informant, the powder is put into all medicines, including antibiotics, is bulk produced and sent to distributors in Mumbai and then to Gujarat, a state in North-western India, and a hub for bulk raw material and API production. The scrap dealer highlighted the adverse effects of the pandemic on cost margins:

*[...] Like raw materials for us with paracetamol for example was ten rupees a kilo is now hundred rupees a kilo... everything has become expensive after Corona (referring to the COVID-19 pandemic)*.

He further commented that India desperately needed raw materials from China during the pandemic, as the market demand for antibiotics was high. In 2019–20 alone, India's reliance on China for API imports was about 63% (James, 2020). However, the COVID-19 pandemic also disrupted the supply chain of APIs from China, pushing India to promote domestic manufacture of APIs in keeping with the broader policy goal of *Atmanirbhar Bharat* or Self-Reliant India.^9^ In 2020, India identified and prioritized the production of 53 raw materials and APIs as part of its “China-plus-one” policy to fill in supply gaps of affordable medicines. The plan included investing $1.3 billion in domestic pharmaceutical producers and potentially reviving state-run companies to ramp up the production of cheap generic drugs (Blankenship, 2022). Imports from China alone grew from INR 23,273 crore in 2021–22 to INR 25,551 crore in 2022–23, while India exported bulk drugs and drug intermediates worth INR 37,853 crore in 2022–23 (Perappadan, 2023). However, this increase in production of antimicrobials meant indigenous pharmaceutical companies faced stiff market competition with a focus on quick production, sales, and environmental clearances (James, 2020). Given that much of the waste and AMR-selection pressure happens during API synthesis, outsourcing antimicrobial production shifts this part of the environmental burden to the producing country. As Bjerke shows, the global API (including antibiotic APIs) demand is fulfilled by countries like China and India, where environmental regulations are weak, labor is cheap, and the workforce is skilled (Bjerke, 2025). India is one of the largest producers and consumers of antibiotics. This, combined with quick production and sales with limited laws on treating effluent waste, puts the burden on environmental and public health in India (Rathinavelu et al., 2024), as was the case during the COVID-19 pandemic.

Manufacturing “the right way” is the responsibility of the pharmaceutical companies, said an official from the State Drug Controller's office in Baddi. The State Drug Controller's office in Baddi sits under the Central Drug Standard Control Office (CDSCO) of the Ministry of Health. It is responsible for testing drug samples and providing No Objection Certificates (NOCs) for the manufacture of medicines, including antibiotics. He continued, “antibiotic manufacturing companies are generally given clearances (referring to NOCs) to produce medicines, but clearances are not given specifically for antibiotics.” Most pharmaceutical companies were unwilling to discuss their antibiotic product formulations or permit factory floor visits, so access to specific antibiotic formulation processes within factories and final products was a challenge. Information about particular antibiotic formulations and the specific companies manufacturing them is confidential and contained in records that require special government permission to access. This meant the specific source and supply of the four selected antibiotics could not be obtained for the Baddi field sites. There is no comprehensive list of companies with the Drug Controller's office that produce antibiotics; instead, the Baddi Drug Controller's office can receive this list from the companies that are involved in making antibiotics.

*Keep in mind human error… even a small percentage of the assay (referring to API %) can be more or less and that can go into the market (referring to sales and consumption), be ingested and make people sick… when people fall ill, then it becomes an issue. Also, it is a matter of finance and profit. If 5% is made from one crore, that is also a big deal… If you want to source cheap raw materials (referring to API %) and make profit… the percentage is less or more and doesn't dissolve (while manufacturing) … goes to sales or market- that's when the problem starts*.

As the official states, profit-making and cost-cutting measures, such as sourcing cheap raw materials, have an impact on the product made available in the market. This, combined with antibiotic-laden effluent waste, creates a hotspot of nullahs, sewage channels, ultimately leading to riverine watercourses, which in this case is the Sirsa. Availability in the market also means increased consumption of medications, including antibiotics, which will make their way into the environment via improper waste disposal and poor sanitation systems. Recent studies, for example, have shown high concentrations of Ciprofloxacin in river water samples from the Sirsa, given its common usage (Dixit et al., 2024).

**Figure 3:**
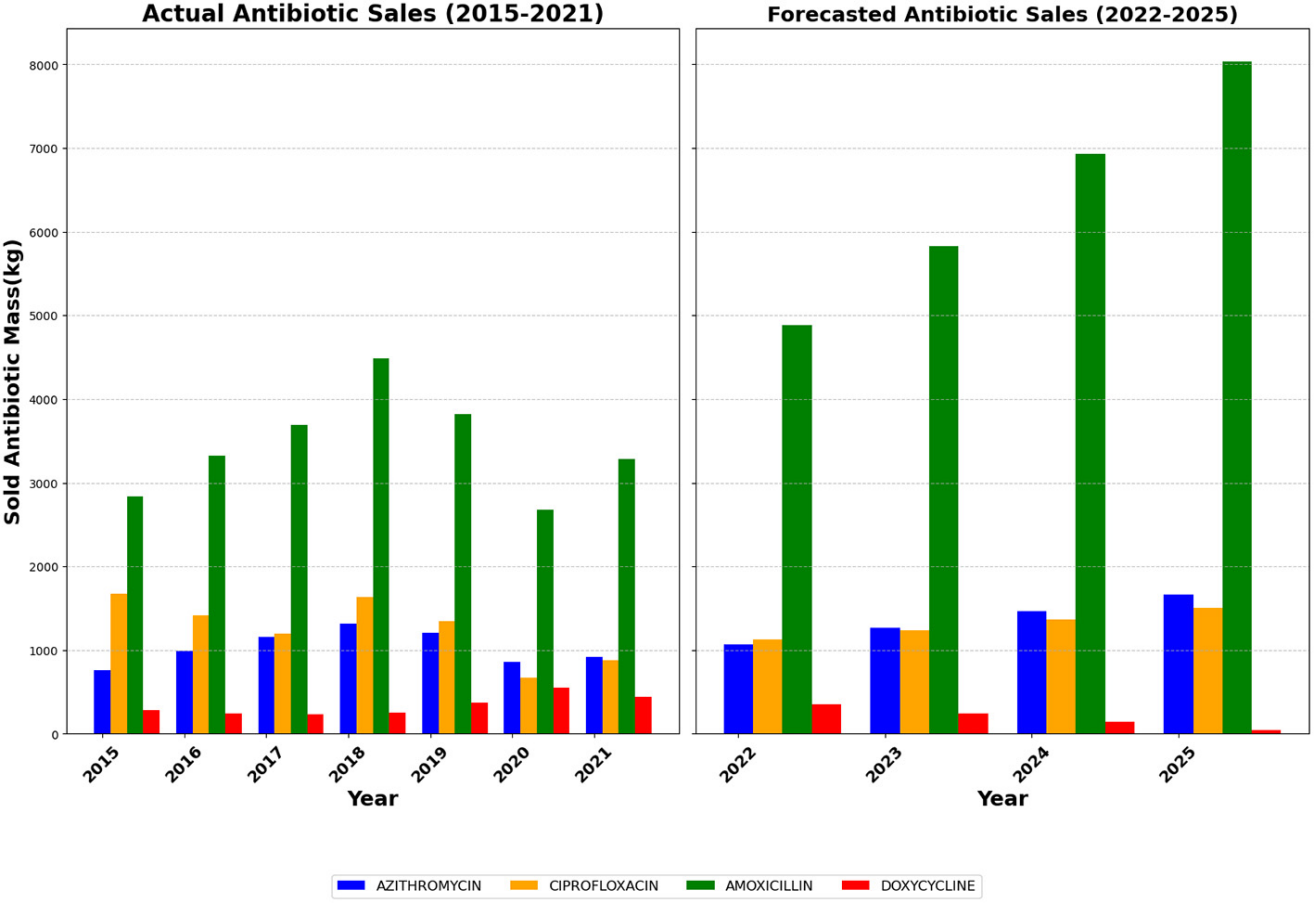
Antibiotic sales data in Himachal Pradesh (2015–2022) with forecasted trends for 2023–2025. Actual sales (2015–2021) show fluctuations with peaks around 2018. Amoxicillin and Azithromycin exhibit a general increase, while Ciprofloxacin and Doxycycline vary more. Forecasted sales (2022–2025) indicate substantial growth, especially for Amoxicillin, which is expected to more than double by 2025. Azithromycin and Ciprofloxacin also show steady increases, while Doxycycline is projected to decline.

Figure 3 (above) presents the historical antibiotic sales data in Himachal Pradesh from 2015 to 2021 and forecasts for 2022–2025. It includes data for four antibiotics—Azithromycin, Ciprofloxacin, Amoxicillin, and Doxycycline—illustrating their trends over time. The historical data (2015–2021) shows noticeable variations in antibiotic consumption, with Amoxicillin consistently leading in sales, followed by Ciprofloxacin, Azithromycin, and Doxycycline. The sales of Amoxicillin saw a peak in 2018, followed by a decline in 2020, likely influenced by disruptions in pharmaceutical production and distribution during the COVID-19 pandemic. Ciprofloxacin and Azithromycin display relatively stable sales trends up to 2019, after which a significant decline is evident in 2020, possibly reflecting shifts in prescribing patterns or temporary supply chain constraints. While consistently the least sold antibiotic, Doxycycline exhibited a steady rise in demand over the years.

The forecasted sales for 2022–2025 suggest a strong recovery, particularly for Amoxicillin, which is expected to experience the most significant increase in sales. Ciprofloxacin and Azithromycin also show a gradual rise in demand, indicating a return to pre-pandemic consumption levels. Doxycycline's sales are projected to stabilize at a relatively lower level. The overall forecast suggests that antibiotic sales will continue their upward trajectory, potentially driven by increased healthcare accessibility, post-pandemic economic recovery, and restored pharmaceutical manufacturing capacity.

The observed fluctuations in antibiotic sales suggest that external factors, such as healthcare policies, global supply chain disruptions (like APIs sourced from China), and disease prevalence (for example, COVID-19 and related symptoms), play a significant role in determining antibiotic consumption. The drop in sales during 2020 aligns with lockdown measures, reduced outpatient visits, and shifts in healthcare priorities that might have temporarily impacted antibiotic prescriptions. Recognizing that this decline might represent an exceptional event rather than a long-term trend, linear trend estimations were employed to project sales for 2023–2025. These projections assume a return to pre-pandemic demand, with sales expected to surpass previous levels due to increasing healthcare access, resumed pharmaceutical operations, and rising antibiotic demand driven by population growth and medical advancements.

### 3.2 Antibiotic consumption, release and discarding into the environment

A senior drug control official in Baddi observed,

*[…] I don't feel very confident to say in this regard whether there has been a decrease in the market after Covid. During Covid, the sales of a particular category was high, even antibiotics because life-saving drugs were in demand…Especially, they [pharma companies] had to provide Azithromycin […] because COVID created a fear that …patients were avoiding going to the OPD (Outpatient Department). OPD visits decreased…Because OPD visits decreased, the market also decreased, and so did the sales. With reduced sales, production also decreased. So, it was a rare phenomenon post-Covid*.

**Figure 4:**
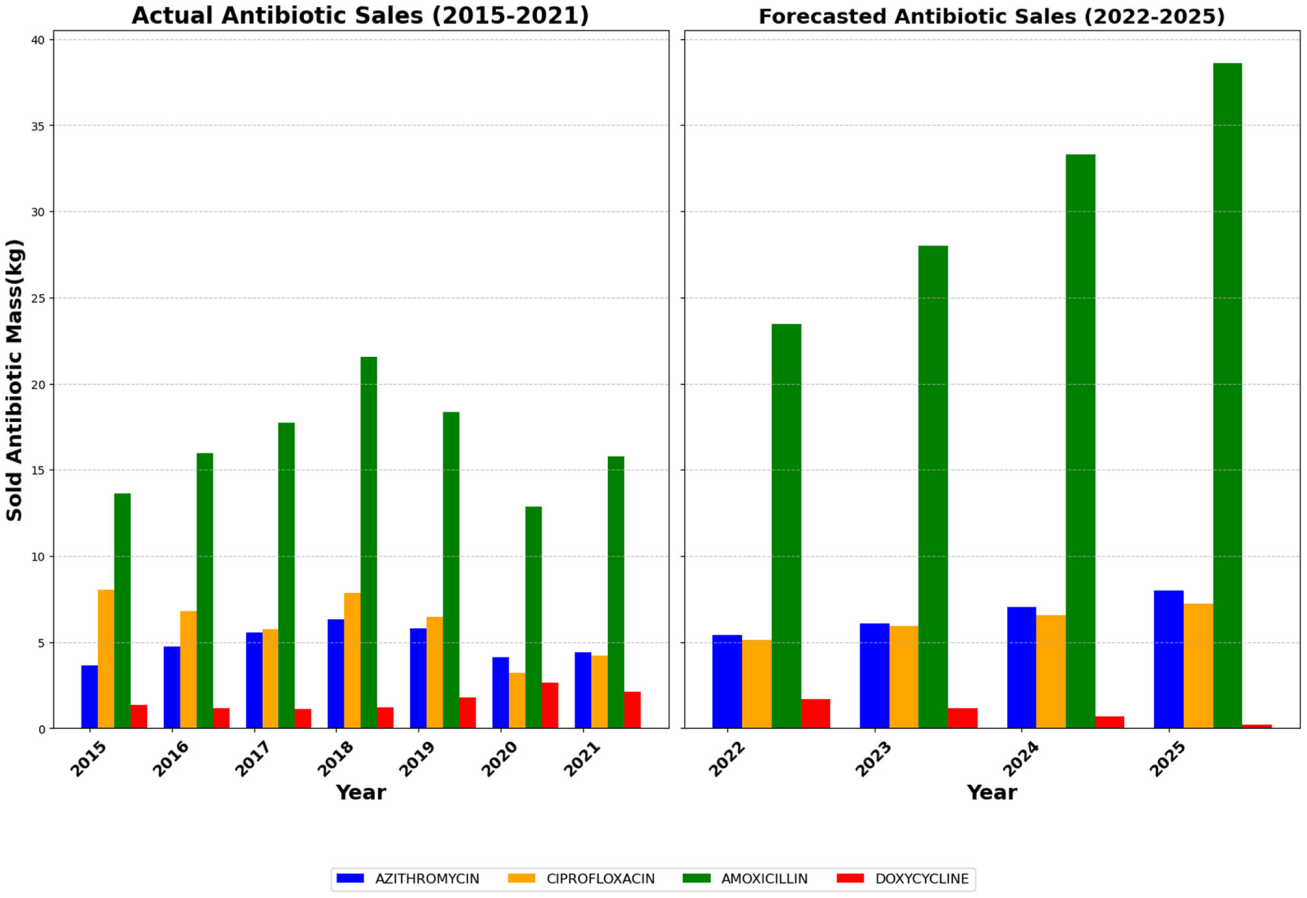
Antibiotic Sales Data in Baddi (2015–2022) with Forecasted Trends for 2023–2025. Actual sales (2015–2021) show fluctuations with peaks around 2018. Amoxicillin and Azithromycin exhibit a general increase, while Ciprofloxacin and Doxycycline vary more. Forecasted sales (2022–2025) indicate substantial growth, especially for Amoxicillin, which is expected to more than double by 2025. Azithromycin and Ciprofloxacin also show steady increases, while Doxycycline is projected to decline.

As the official highlighted, the consumption of antibiotics amongst communities in Baddi did not entirely cease during or after the pandemic, despite the 2020 dip in antibiotic sales (see Figure 4) that may be accounted for by the decrease in OPD visits described by our informant. As elsewhere in India and other low and middle-income countries, over-the-counter purchasing of antibiotics without a prescription is common in Baddi, and the pandemic generated novel ways of interpreting symptoms related to sore throat, cold, and fever, leading to self-medication with strong antibiotics like amoxicillin and azithromycin (Shrestha et al., 2022).

Figure 4 is derived by proportionally scaling the Himachal Pradesh sales data according to the population of Baddi. Given Baddi's prominence as a key pharmaceutical hub, this estimation provides a localized perspective on antibiotic consumption patterns within the region. The assumed proportionality allows for a more precise understanding of regional pharmaceutical sales, offering insights into demand fluctuations that might differ from broader state-wide trends. The estimated figures indicate similar fluctuations observed at the state level, with Amoxicillin remaining the most consumed antibiotic, and the COVID-19-related dip in sales reflected proportionally.

Baddi has one civil hospital, which is government-run and provides free treatment for the public. The hospital is located not far from the city center, and the staff also run weekly mobile clinics that the Glenmark Foundation, the Corporate Social Responsibility arm of Glenmark Pharmaceuticals, a leading global pharmaceutical company with a significant presence in Baddi, sponsors. We accompanied Dr. Anu, a general consultant at the civil hospital, on a mobile clinic visit to Nalagarh (a municipality close to Baddi). The mobile unit carries basic medication in tin boxes (kept in the back of the car), and the driver arranges a table and a chair in a designated open space, which remains the same every week. Dr. Anu's assistant opens the boxes, collects the *parchiyan* (previous prescriptions), asks patients to wait in order of the *parchiyan*, and then calls out each name in turn for Dr. Anu to check. On one such visit, we asked her about the prevalence of disease and any unnecessary prescription of antibiotics:

*A child came to me with chest congestion and cough. Someone had prescribed azithromycin so that symptoms subside soon. It's not required for mild cough… it's a strong ATB (antibiotic). I gave amoxicillin for 5 days. The mother said that the child is fine. See if you give strong ATBs…. The child is bound to develop resistance (referring to the microbe developing resistance)*.

The doctor goes on to tell us that Baddi has a prevalence of chronic patients with diabetes and hypertension who continue “popping” antibiotics to “feel better” or use previous prescriptions for instant relief and stop taking them a day or two after they start “feeling better.” A private pharmacist who has a shop near the civil hospital added,

[…] *Suppose if a patient comes to me for medicine or antibiotics and I refuse to give him antibiotics without doctor's prescription, then that patient will go to the next pharmacy shop and take the same antibiotics and medicine from there. Therefore, it will not make any difference whether I give antibiotics or refuse them. Because that patient will take antibiotics from some other pharmacy. Therefore, I believe that when doctors prescribe antibiotics to a patient in any government or private hospital or clinic, they should not prescribe strong antibiotics in the beginning. For example, many times a patient has a cough and is cured by amoxicillin 500, but many times the doctor prescribes an injection of iv ceftriaxone BD. Due to this amoxicillin will not affect the patient (referring to possible development of AMR) the next time he falls ill*.

The pharmacist further added that antibiotics are given only by prescription at the shop, but some patients come to the shop without a doctor's consultation or a diagnostic test report. It is common for some patients to ask for antibiotics suggested by their “village doctor,” who is based in their home state of Uttar Pradesh or Bihar (two other populous states in North India), to get medicines from the local pharmacy. A popular unqualified “doctor” at a slum in Baddi provided insight into how local slum (*juggi*) dwellers obtain medication. He has a diploma in pharmacy (popularly called DPharma), is from Uttar Pradesh and has his home in a *juggi* near a popular pharmaceutical company factory. The *juggi* doctor has converted the front part of his home into a consultation room where he sees local patients. A mother walks in with her daughter in her arms and says that her child has a fever. The doctor nods and, telling me that the child is 9 years old, gives the mother a thermometer to place under its arm:

*We give medicines according to the age of the child… I told you this earlier and we give a tablet or syrup accordingly. [As he is talking to us, the doctor prepares two separate pudiyas of medicines which each contain] Paracetamol - 150 mg for fever, Cetirizine – half (of the tablet) for antifungal [in fact, it is an antihistamine], Ranitidine- for gas and acidity, Leukuf- for cough, Amoxicillin- antibiotic for cough* **. **

**Figure 5:**
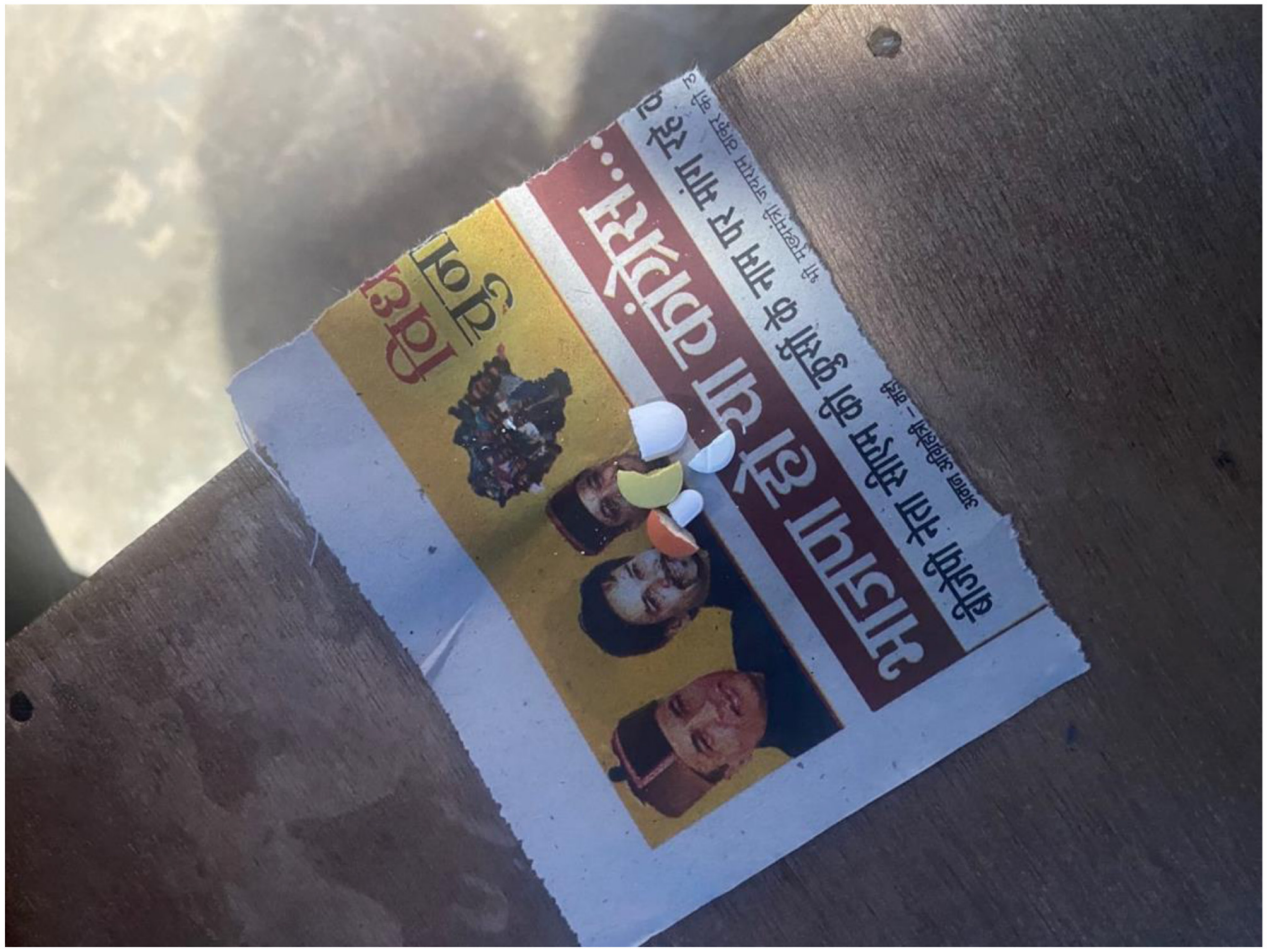
Open medicine *pudiya* (photo by Amishi Panwar 2023).

Each packet (*pudiya*, see Figure 5) is a dose of medication, one to be taken in the morning and the other in the evening. The doctor takes INR 50 for the *pudiyas*, which have been prescribed for fever and smiling, and gives back 10 rupees so the child can buy a toffee.

Most of the residents in the slum are laborers and their families working in textile, pharmaceutical, and steel factories located in and around Baddi, or are daily wage earners working as masons or carpenters. Some are daily wage laborers with no steady income. Given their financial constraints, local providers such as the practitioner quoted above commonly provide medicines for no more than 3 days at a time, even for an antibiotic course that should be taken for 5 days. Such practices could lead to ineffective treatment due to underdosing, not killing the pathogens, or the pathogens within the body developing AMR over time (Shrestha et al., 2022; Green et al., 2023; Cousins, 2018).

**Figure 6:**
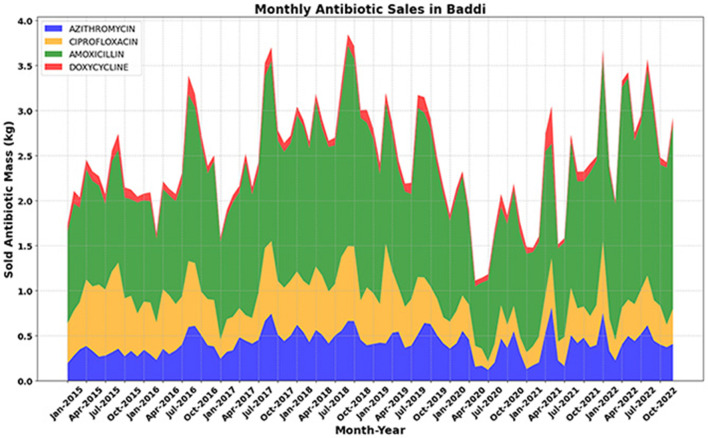
Monthly antibiotic sales data in Baddi (2015–2022), where a clear drop is observed in the first half of 2020. For Amoxicillin and Doxycycline, an increasing trend in sales is evident from 2015 to 2018. For azithromycin and ciprofloxacin, there was a flatter trend from 2015 to 2019, but a similar noticeable drop in sales was observed in mid-2019 and 2020.

Figure 6 (below) provides a more granular view of the monthly antibiotic sales data in Baddi from 2015 to 2022, allowing for a detailed examination of consumption dynamics. The monthly trend analysis reveals a steady increase in Amoxicillin and Doxycycline sales from 2015 to 2018, while Ciprofloxacin and Azithromycin demonstrate a more stable pattern over the same period. However, a substantial drop in sales is observed in early 2020, coinciding with the onset of the COVID-19 pandemic. This decline is most evident for Ciprofloxacin and Azithromycin, possibly reflecting changes in prescribing behavior or disruptions in pharmaceutical supply chains. Interestingly, Amoxicillin and Doxycycline, which had shown an upward trend before 2018, exhibit resilience post-pandemic, continuing their rise in demand.

Another medical practitioner in the same *juggi* also has a DPharma and is learning from his elder brother as they practice in the consultation room located in front of their house, where slum dwellers come to him with their health issues. The medication (the practitioner refused to share the exact dosage) is again given in combination, i.e., a mixture of antibiotics, antihistamines, or paracetamol to treat a stomachache. The practitioner stated,

*The first thing is that there is hardly any leftover. We get 5 days course. We complete the course. Because people are very serious with the medicine course. They don't want to throw medicines. If they don't get certain medicines and they get it from outside. They are expensive. Nobody wants to throw it away. So, they take it. Very less people throw medicines […] most of the people throw it in drainage if such situation (referring to people feeling well in 3 days after taking medication for a 5-day course) is there*.

**Figure 7:**
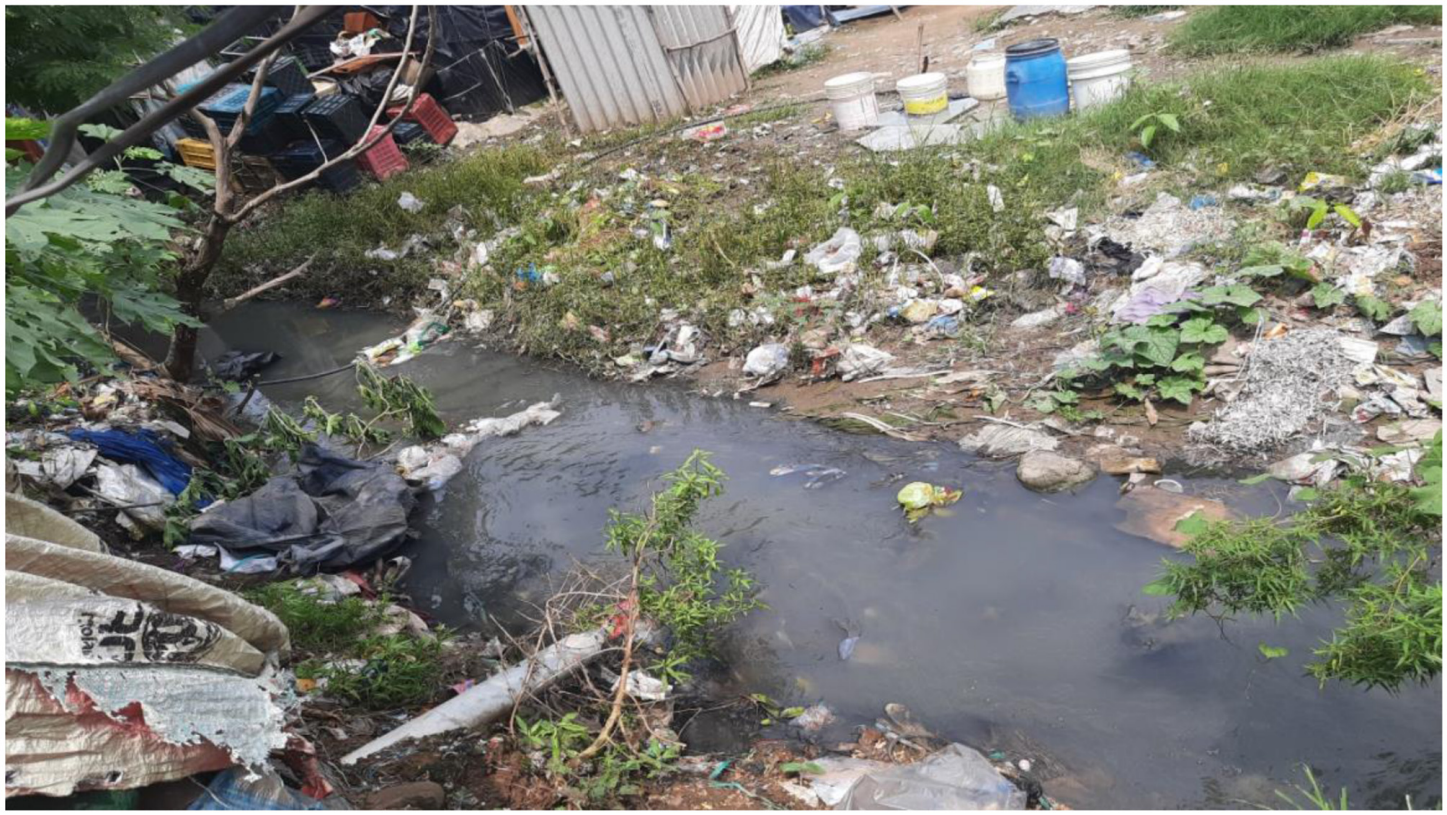
Open drainage with wastewater from kitchens, toilets, and washing area in the *juggi* merging (not visible in photo) with the Sirsa river ahead (photo by Gian Singh Negi 2023).

If unused medicines are thrown into the open drainage channels or if they're “left over and expired, a hole is dug in the ground and they are buried in the ground so that children won't find or pick and eat them,” as another slum dweller mentioned. These then become a point of environmental entry of antimicrobials via the sewage water from the *juggi* to the nearest water body and then into the river Sirsa or the soil (see Figure 7), leading to environmental antibiotic pollution. Compared with antibiotic environmental pollution from industry and sewage, however, this may be a relatively minor source of contamination.

Another slum dweller, Guddu, whose *juggi* was situated not far from the open drainage (as seen in Figure 7), stated,

*[…] everything (toilets) is near the drainage. Some people make it like a group (toilet). Some people make it on their own (toilets). Like I am alone, so I have made my own separate bathroom […] toilet waste goes into open drainage […] it is not covered…no septic tank…clogged drain is the main reason for pollution. Nobody comes to clean it. It has not been cleaned for two years. I haven't seen it cleaned. If it rains heavily then it gets washed away. Otherwise, it is clogged here most of the times*.

Guddu is in his early twenties, and his statement above highlights the state of open toilets and waste being clogged close to living quarters. As seen in Figure 7 (on the top right corner where plastic buckets are placed for storing water), water for drinking and cooking is sourced from a borewell in a nearby locality. The landlord of this slum has provided a drinking water facility in his area of residence. The pipe carrying drinking water runs through the slum, and in some cases, it originates from within the clogged drainage stream. The clogged drainage stream consists of water from domestic usage, excretion, washing clothes and utensils, and bathing. These practices contribute to another source of environmental antibiotic pollution, primarily through human excretion and the use of topical antibiotics, which mix with open drainage and eventually flow into the river Sirsa.

### 3.3 Treatment and disposal of antibiotic manufacturing waste into the environment

Baddi is home to many pharmaceutical formulation companies with headquarters in Chandigarh and New Delhi. Some of these companies have leased out the manufacture and production of antimicrobials to third-party companies in Baddi. In one such instance, we met a pharma company executive who has many pharmacies based in the National Capital Region (NCR) with manufacturing leased to a company in Baddi. The informant spoke extensively about manufacturing practices that could present potential pathways for antimicrobial environmental exposure. Some pharmaceutical factories have their dedicated effluent treatment plants (ETPs), and international industry bodies have issued guidance advocating for manufacturers to limit their environmental emissions of antimicrobials (SIWI, 2023). The AMR Industry Alliance (AMRIA) initially issued an Antibiotic Manufacturing Standard in 2022, which “requires that the manufacturer of an antibiotic must have an effective environmental management system and that the antibiotic's Predicted No-Effect Concentrations, or the level at which a substance will not have an adverse effect on its environment, are met” (Antibiotic Manufacturing Standard, 2025). This Standard was updated in May 2025 after the publication of the WHO's guidance on wastewater and solid waste management for manufacturing of antibiotics in September 2024, which calls for transparency, stringent reporting and internal audits (World Health Organization, 2024). The AMRIA has since maintained a list of derived antibiotic PNECs (Predicted No-Effect Concentrations) following the WHO guidelines and has further made recommendations for effective wastewater treatment, record keeping, and monitoring (Antibiotic Manufacturing Standard, 2025). However, such measures require additional investment. Heavy investment in antibiotic production significantly impacts the entire supply chain, encompassing cost-cutting measures, negotiations over production pricing, and adherence to minimal regulatory requirements (Hamill et al., 2023). In some instances, this also includes the disposal of antibiotics and related products. As the executive further highlights,

*[…] ok that person (referring to the third-party manufacturing unit) will say… I will give it (medicines) to you in ten rupees (referring to 10 INR). We will say “don't do it in ten rupees, do it in nine and a half rupees or nine rupees” […] like they will negotiate, right. So, that… unfortunately the manufacturer as a result is also you know is on thin margins. So, there is cost cutting pressures on him also. So, they take theis easy way out where you know if they have to dispose things in certain way they may not be doing […]*

The implication here is that low profit margins may lead to cost-cutting and improper disposal of antibiotic production waste. Even where profit margins are not threatened, effective treatment of antibiotic waste is difficult to ensure, and antibiotic residues are not currently monitored. All industries in Baddi are required to send their waste for treatment to the Central Effluent Treatment Plant (CETP),^10^ where waste is treated and eventually released into the Sirsa River. We visited one liquid waste treatment site and two solid waste treatment plants (one catering to domestic waste and the second catering to industrial waste). The solid waste treatment plant located on the outskirts of Baddi is surrounded by lush greenery and has a vast area cleared for landfills. The landfills are designed by one of India's premier technology institutes to prevent any waste leakage. However, given that antibiotic residues are not tested before being dumped into the landfill, the extent of the impact on the environment, reaction with other waste, and transfer of ARGs via leaching and leakage (Zhang et al., 2022) is unknown. To trace industrial and domestic waste disposal, interviews were conducted with solid and liquid waste treatment plant heads to gauge the treatment and release of antibiotic residues.

*ATB residues are not tested… I will explain everything else…. All waste is treated with lime and cement and buried in landfills*. [Biotech Lead, Solid Waste Management Plant]

This informant states that none of the three treatment plants are specifically designed to treat antibiotic residues or separate antibiotic medicines from domestic waste in the Baddi area. “Domestic waste is sorted by size, medicines are not specifically sorted,” said the solid waste treatment plant head. The solid waste treatment plant, located near the CETP in Baddi, has accumulated a substantial amount of domestic waste, including food, metals, and plastic, on site. None of these facilities has active antibiotic residue testing. Another official at the liquid waste treatment plant, who is a chemist by training, mentioned that they operate under strict guidelines of the State Pollution Control Board (SPCB), Baddi, regarding effluent treatment. Pollution Control Board and other government guidelines for hazardous waste do not currently include specific guidance to regulate antibiotic residues.

National organizations, including the Center for Science and Environment (CSE) and the National Green Tribunal (NGT) have highlighted the need to enhance environmental monitoring, strengthen waste management practices, and strengthen regulatory frameworks regarding antibiotic waste in India. A 2022 article in the CSE's newsletter, *Down to Earth*, presented evidence that pharmaceutical companies were responsible for polluting the Sirsa River with high concentrations of antibiotic residues via wastewater discharge, suggesting that this was higher than from indirect sources, including antibiotic residues via human and animal feces or the disposal of unused medicines (Bhati, 2022). Water samples from industrial areas around Baddi, which were tested as part of the ResPharm project between 2019 and 2023, also showed the presence of antibiotics (e.g., sulfamethoxazole, ciprofloxacin, rifampicin) and other APIs such as antihistamine cetirizine (unpublished data). *Ground Report*, a digital news platform for health, gender, and environmental issues, recently reported that the National Green Tribunal has called for comprehensive responses from all states and Union Territories, requesting disclosure of the compliance status of pharmaceutical companies with environmental norms and regulatory guidelines (Ground Report, 2024).

## 4 Discussion: antimicrobial pollution and mitigation strategies

Antibiotic sales data from Baddi and Himachal Pradesh offer important insights into local consumption patterns. Sales volumes of antibiotics like Azithromycin and Amoxicillin indicate high demand, possibly driven by issues identified through our qualitative work, such as prescription practices, over-the-counter antibiotic availability, and high self-medication rates. The data shown in Figures 3, 4, and 6 collectively reveal trends in antibiotic sales in Himachal Pradesh and Baddi. While historical data show fluctuations influenced by external factors like the pandemic, forecasted trends suggest a strong recovery. These insights can help policymakers, healthcare professionals, and pharmaceutical manufacturers predict future demand, improve production, and promote sustainable antibiotic distribution to support SDG 12 (responsible consumption, production, and waste) and SDG 8 (sustainable economic growth). Understanding these patterns is essential for several reasons.

*Public Health Planning*: Information on antibiotic consumption is vital for creating targeted antibiotic stewardship programs. In 2015, the WHO introduced a Global Action Plan (GAP) emphasizing the importance for member states to prioritize antimicrobial stewardship programs (AMSP) in their National Action Plans (NAPs), as an intervention to reduce overuse of antibiotics and resistance (Walia et al., 2019). For instance, our findings suggest that high consumption of certain antibiotics may be caused by over-prescription or self-medication, particularly among vulnerable populations with limited healthcare access (Adebisi and Ogunkola, 2023), especially during the COVID-19 pandemic when antibiotics were overused despite their ineffectiveness against viruses. Structural barriers, such as public healthcare inefficiencies and financial and time constraints, create alternative access points to antibiotics, contributing to antimicrobial resistance (Green et al., 2023; Adebisi and Ogunkola, 2023). This study underscores structural inequalities and shows how antibiotic resistance may be more common in vulnerable groups, aligning with SDG 10, which aims to reduce inequalities—a critical aspect within the antibiotic supply chain. This disparity highlights the necessity for better stewardship programs to reduce unnecessary antibiotic use and slow resistance development. Notably, shifts in sales patterns during COVID reveal how wider healthcare and epidemiological disruptions impact antibiotic consumption, emphasizing the importance of strengthening antibiotic stewardship (Pierce and Stevens, 2021). Additionally, tracking antibiotic consumption trends can support public health campaigns aimed at educating both the public and healthcare professionals about the risks of resistance and the importance of responsible antibiotic use.

*Environmental Impact Assessment*: Regularly using certain antibiotics like Amoxicillin, Ciprofloxacin, Azithromycin, and Doxycycline requires further environmental monitoring. As our study shows, the high sale of antibiotics like Amoxicillin and Ciprofloxacin also means a significant presence of these antibiotics in the environment, thereby polluting the environment and increasing the risk of AMR. Our study identifies areas with potentially significant antibiotic runoff, suggesting specific effluent wastewater ways near pharmaceutical plants and sewage wastewater systems in slum communities in Baddi for targeted testing. This strategy helps identify points of environmental entry of antimicrobials and evaluate the risk of antibiotic resistance in the environment, thereby informing mitigation strategies to address Target 3.9 (Reduce the number of deaths and illnesses from hazardous chemicals and air, water, and soil pollution and contamination). Mitigation strategies could include a sustainable method of treating such wastewater and sewage water systems, potentially making it a resource for irrigation, and thereby strengthening local food production systems.^11^ Carter highlights the importance of a “circular economy” whereby waste is designed to be eliminated and used within a “closed loop” system (Carter et al., 2024). Furthermore, the multinational pharmaceutical company managers based in Baddi claim to treat all the effluent wastewater and use it for watering company-run gardens and for cleaning purposes. Some medium and small-scale pharma companies are required to send the wastewater for treatment to CETPs (Sinha, 2017). As our data suggest, given that the ETPs and the CETP in Baddi are not designed to eliminate antibiotic residues, questions of limiting the usage of contaminated and recycled wastewater with residual APIs remain.

*Policy and Regulation*: Insight into antibiotic sales and consumption patterns can inform policy decisions, specifically NAPs, drug regulation and control (specifically antibiotics) and wastewater treatment guidelines in India. For instance, the fluctuating antibiotic sales during health crises like the COVID-19 pandemic underscore the need for robust regulatory frameworks that can adapt to sudden shifts in drug use patterns and still “allow equitable access to effective antibiotics” (Ren et al., 2022). Our study highlights the critical need for stricter environmental stewardship programmes across the entire supply chain, emphasizing the accountability of international partners who outsource production to India. Policies that require stricter sales monitoring, prescription practices, and comprehensive waste management from pharmaceutical plants could be crucial. For example, the WHO guidance has called for a check on steps from the manufacturing of active pharmaceutical ingredients (APIs) and formulation into finished products, including primary packaging. Keeping in mind the WHO's aim of achieving SDG 3 (United Nations, 2024), which aims to achieve healthier environments and healthy lives to prevent almost a quarter of the global disease, pollution prevention and control from municipal systems, manufacturing sites, healthcare facilities, and agri-food systems also must be monitored (World Health Organization, 2024). The AMRIA has cited risk assessment as a critical step of curbing AMR in the environment, including minimizing API loss, segregation, and pretreatment of waste (Antibiotic Manufacturing Standard, 2025), which is a step toward regulating the antibiotic supply chain.

## 5 Conclusion

This study highlights the pressing need for a synchronized response at both the state and national levels to augment environmental monitoring, enhance waste management practices, and fortify regulatory frameworks for India's antibiotic manufacturing and waste disposal.

The forecasted data suggesting a continued upward trajectory in antibiotic sales underscores the urgency for coordinated responses at both state and national levels. These responses should focus on enhancing environmental monitoring efforts, improving waste management practices, and strengthening regulatory frameworks to address the environmental implications of antibiotic production, consumption, and disposal in India. By understanding local consumption patterns, stakeholders can implement targeted interventions to promote the rational use of antibiotics, enhance regulatory frameworks, and protect both public health and the environment from the adverse effects of antibiotic pollution and resistance.

To establish the link between antibiotic consumption and environmental pollution, it is imperative to consider factors such as local sanitation systems and the extent of municipal provision, particularly in slum areas. The discharge of effluents containing antibiotics into local rivers can contribute to environmental contamination. These factors contribute to the broader issue of antibiotic pollution, further exacerbating the challenge of antimicrobial resistance. In conclusion, our study highlights risk pathways linked to antibiotic manufacture and consumption patterns, which will have direct implications on public health and the environment. The noted shifts in antibiotic usage during the COVID-19 crisis underscore the pressing need to strengthen antibiotic stewardship across all levels of healthcare provision to support environmental protection. This reinforcement should aim not only to address current misuse but also to prepare for future public health emergencies.

## Data Availability

The raw data supporting the conclusions of this article will be made available by the authors, without undue reservation.

## Footnotes

^1^The One Health framework is a multi-sectoral, multi-disciplinary and collaborative approach working towards a sustainable health delivery system. “A holistic approach validated by international organizations, the World Health Organization (WHO), World Organization for Animal Health (OIE), and the Food and Agriculture Organization (FAO) of the United Nations to work in collaboration for the welfare of the whole ecosystem through human-animal-plant-environment interaction (Raut et al., 2023).

^2^For more information on all the Sustainable Development Goals listed, see United Nations (2024).

^3^For more information on the projects, see Natural Environment Research Council (2020).

^4^For more information on project ResPHARM, see ResPHARM (2024).

^5^For more information on Project AMRflows, see AMRflows (2024).

^6^The coordinates for the Baddi study are Latitude −30.939367, Longitude −76.798499; Latitude −30.931976, Longitude −76.827186; Latitude −30.923322, Longitude −76.808089; Latitude −30.92385, Longitude −76.83294; Latitude −30.92381, Longitude −76.83319.

^7^The questionnaire used for conducting interviews was developed only for this study has been attached as a Supplementary material.

^8^The “red category” includes petrochemicals, pharmaceuticals, sugar, paper and pulp, nuclear power plants, organic chemicals, fertilizers, firecrackers, etc. For information on the color-coded categorization, see the draft “Classification of Industrial Sectors into Red, Orange, Green, and White Categories: A Tool for Progressive Environmental Management” published by the Central Pollution Control Board, New Delhi (July 2023; ACMA, 2023).

^9^For more information on the *Atmanirbhar Bharat* Campaign, see Invest India's overview (Invest India, 2024).

^10^For more information on the working of CETP in Baddi, which works under the purview of the Himachal Pradesh Pollution Control Board (HPPCB; CETP–Baddi Infra, 2019).

^11^For more information, see the webpage, “Can waste be a resource?” (Carter, 2020).

## References

[B1] ACMA (2023). The Automotive Component Manufacturers Association of India. Available online at: https://www.acma.in/uploads/docmanager/Draft_Classification%20of%20Industrial%20Sectors%20into%20Red (accessed July 18, 2024).

[B2] Adebisi Y. A. Ogunkola I. O. (2023). The global antimicrobial resistance response effort must not exclude marginalised populations. Trop. Med. Health. 51:33. 10.1186/s41182-023-00524-w 37287083 PMC10245439

[B3] AMRflows (2024). AMRflowsUK-India project: antimicrobials and resistance from manufacturing flows to people: joined up experiments, mathematical modelling and risk analysis. *Bham.ac.uk*. Available online at: https://more.bham.ac.uk/amrflows/ (accessed February 16, 2024).

[B4] Antibiotic Manufacturing Standard (2025). Antibiotic Manufacturing Standard: Minimizing the Risk of Developing Antibiotic Resistance and Aquatic Ecotoxicity in the Environment Resulting from the Manufacture of Human Antibiotics. Available online at: https://www.amrindustryalliance.org/wp-content/uploads/AMR-Industry-Alliance-Manufacturing-Standard.pdf (Accessed August 4, 2025).

[B5] Barathe P. Kaur K. Reddy S. Shriram V. Kumar V. (2024). Antibiotic pollution and associated antimicrobial resistance in the environment. J. Hazardous Mater. Lett. 5:100105. 10.1016/j.hazl.2024.100105

[B6] Bhati D. (2022). A third of monitored antibiotic manufacturers in Himachal found to be polluting. Down to Earth. Available online at: https://www.downtoearth.org.in/pollution/a-third-of-monitored-antibiotic-manufacturers-in-himachal-found-to-be-polluting-81607 (Accessed July 2, 2024).

[B7] Bjerke L. (2022). Antibiotic geographies and access to medicines: tracing the role of India's pharmaceutical industry in global trade. Soc. Sci. Med. 312:115386. 10.1016/j.socscimed.2022.115386 36182675 PMC9489990

[B8] Bjerke L. (2025). Antibiotics in the environment: molecularisation, drug resistance and pharmaceutical pollution in India. BioSocieties. 20, 551–578. 10.1057/s41292-025-00353-6

[B9] Blankenship K. (2022). India, Hoping to Challenge Chinese Dominance, Plans API Production Push: Report. Available online at: https://www.fiercepharma.com/manufacturing/india-hoping-to-challenge-chinese-dominance-plans-drug-ingredient-production-push (Accessed July 21, 2024).

[B10] Cameron A. Connolly J. Esiovwa R. Henriquez F. L. Hursthouse A. Mukherji S.. (2025). ‘Mind the gaps': stakeholder perspectives on addressing antimicrobial resistance in the environment in the Indian context. Global Health Action 18:2491200. 10.1080/16549716.2025.2491200 40308153 PMC12046612

[B11] Carter L. (2020). Chemical Pollution Knows No Borders. Available online at: https://spotlight.leeds.ac.uk/world-changers/chemical-pollution/#group-section-Can-waste-be-a-resource-lof0cCtyYO (Accessed July 27, 2024).

[B12] Carter L. J. Dennis S. Allen K. McKenna P. Chen X. Daniell T. J.. (2024). Mitigating contaminant-driven risks for the safe expansion of the agricultural–sanitation circular economy in an urbanizing world. ACS Es&T Water 4, 1166–1176. 10.1021/acsestwater.3c00803 38633372 PMC11019536

[B13] CETP–Baddi Infra (2019). Available online at: https://baddiinfra.org/cetp/ (Accessed July 11, 2024).

[B14] Cornell Law School (2014). 21 CFR § 203.3 - Definitions. LII/Legal Information Institute. Available online at: https://www.law.cornell.edu/cfr/text/21/203.3 (Accessed April 22, 2024).

[B15] Cousins D. (2018). Patients are being underdosed: we need new guidance on small-volume drug infusions. Pharm. J. 10, 356–357. 10.1211/CP.2018.20205779

[B16] Dixit A. Pandey H. Rana R. Kumar A. Herojeet R. Lata R.. (2024). Ecological and human health risk assessment of pharmaceutical compounds in the Sirsa River of Indian Himalayas. Environ. Pollut. 347:123668. 10.1016/j.envpol.2024.123668 38442820

[B17] Green D. L. Keenan K. Fredricks K. J. Huque S. I. Mushi M. F. Kansiime C.. (2023). The role of multidimensional poverty in antibiotic misuse: a mixed-methods study of self-medication and non-adherence in Kenya, Tanzania, and Uganda. Lancet Global Health 11, e59–68. 10.1016/S2214-109X(22)00423-5 36521953

[B18] Ground Report (2024). NGT Issues Notices to States, UTs Regarding Pharmaceutical Pollution. Ground Report. Available online at: https://www.groundreport.in/pollution/ngt-issues-notices-to-states-uts-regarding-pharmaceutical-pollution-4549529 (Accessed June 24, 2024).

[B19] Hamill H. Hampshire K. Vinaya H. Mamidi P. (2023). Insights from a qualitative study of the procurement and manufacture of active pharmaceutical ingredients in India. BMJ Global Health 6:e011588. 10.1136/bmjgh-2022-011588 37197796 PMC10201222

[B20] Invest India (2024). Atmanirbhar Bharat Abhiyaan | Self-reliant India Campaign. About Us - Invest India. Available online at: https://www.investindia.gov.in/about (Accessed July 19, 2024).

[B21] James T. (2020). Bulk Drug Industry in India: Challenges and Prospects RIS Discussion Paper Series. Available online at: https://ris.org.in/sites/default/files/Publication/Discussion%20Paper%20259%20T%20C%20James.pdf (Accessed July 2, 2024).

[B22] Kraemer S. A. Ramachandran A. Perron G. G. (2019). Antibiotic pollution in the environment: from microbial ecology to public policy. Microorganisms 7:180. 10.3390/microorganisms7060180 31234491 PMC6616856

[B23] Kümmerer K. (2009). Antibiotics in the aquatic environment–a review–part I. Chemosphere 75, 417–434. 10.1016/j.chemosphere.2008.11.086 19185900

[B24] Li L. Zhang T. (2023). Roadmap to tackle antibiotic resistance in the environment under the One Health framework. mLife 2, 224–228. 10.1002/mlf2.12078 38817813 PMC10989945

[B25] Maps of India (2025a). Himachal Pradesh District Map. Available online at: https://www.mapsofindia.com/maps/himachalpradesh/himachalpradesh.htm (Accessed September 1, 2025).

[B26] Maps of India (2025b). Political Map of India. Available online at: https://www.mapsofindia.com/maps/india/india-political-map.htm (Accessed September 1, 2025).

[B27] Martinez J. L. (2009). Environmental pollution by antibiotics and by antibiotic resistance determinants. Environ. Pollut. 157, 2893–2902. 10.1016/j.envpol.2009.05.051 19560847

[B28] Natural Environment Research Council (2020). India-UK Tackling AMR in the Environment from Antimicrobial Manufacturing Waste (AMR-India) – Strengthening Surveillance Capacity and Frameworks Indiaukamrenvironment.org. Available online at: https://indiaukamrenvironment.org/ (Accessed February 4, 2024).

[B29] Panwar A. Uluseker C. Negi G. S. Lambert H. (2024). Mapping Environmental Exposure and AMR from antimicrobial production, consumption and disposal in Baddi, India [Preprint]. Research Square. Available online at: https://www.researchsquare.com/article/rs-4938099/v1 (Accessed July 2, 2025).

[B30] Perappadan B. S. (2023). India's import of Active Pharmaceutical Ingredients and Key Starting Material from other countries including China grew: Ministry. The Hindu. Available online at: https://www.thehindu.com/news/national/indias-import-of-active-pharmaceutical-ingredients-and-key-starting-material-from-other-countries-including-china-grew-ministry/article67621117.ece (Accessed July 20, 2024).

[B31] Pierce J. Stevens M. P. (2021). COVID-19 and antimicrobial stewardship: lessons learned, best practices, and future implications. Int. J. Infect. Dis. 113, 103–108. 10.1016/j.ijid.2021.10.001 34624517 PMC8491953

[B32] Pruden A. Pei R. Storteboom H. Carlson K. H. (2006). Antibiotic resistance genes as emerging contaminants: studies in northern Colorado. Environ. Sci. Technol. 40, 7445–7450. 10.1021/es060413l 17181002

[B33] Rathinavelu S. Uluseker C. Sonkar V. Thatikonda S. Nambi I. M. Kreft J.-U. (2024). Mapping the scarcity of data on antibiotics in natural and engineered water environments across India. Front. Antibiot. 3:1337261. 10.3389/frabi.2024.1337261 39816266 PMC11732091

[B34] Raut J. Joshi A. Mudey A. Mehendale A. M. (2023). The past, present, and future of one health in india: a narrative review. Cureus 15:e44992. 10.7759/cureus.44992 37829943 PMC10564975

[B35] Ren M. So A. D. Chandy S. J. Mpundu M. Peralta A. Q. Åkerfeldt K.. (2022). Equitable access to antibiotics: a core element and shared global responsibility for pandemic preparedness and response. J. Law Med. Ethics 50, 34–39. 10.1017/jme.2022.77 36889350 PMC10009365

[B36] ResPHARM (2024). Available online at: https://respharm.net/ (Accessed February 16, 2024).

[B37] Rizzo L. Manaia C. Merlin C. Schwartz T. Dagot C. Ploy M. C.. (2013). Urban wastewater treatment plants as hotspots for antibiotic-resistant bacteria and genes spread into the environment: a review. Sci. Total Environ. 447, 345–360. 10.1016/j.scitotenv.2013.01.032 23396083

[B38] Shrestha A. B. Aryal M. Magar J. R. Shrestha S. Hossainy L. Rimti F. H.. (2022). The scenario of self-medication practices during the covid-19 pandemic; a systematic review. Ann. Med. Surg. 82:104482. 10.1016/j.amsu.2022.104482 36059596 PMC9419440

[B39] Sinha R. (2017). How Baddi pharma waste can make your medicines ineffective. Down to Earth. Available online at: https://www.downtoearth.org.in/waste/bitter-medicine-57879 (Accessed July 15, 2024).

[B40] SIWI (2023). Responsible Antibiotics Manufacturing Platform (RAMP) Framework | SIWI- Leading Expert in Water Governance. Available online at: https://siwi.org/publications/ramp-framework/ (Accessed August 7, 2024).

[B41] Subramanian S. (2023). Pharma and healthcare for India@100: a century of change on the horizon. www.ey.com. Available online at: https://www.ey.com/en_in/health/pharma-and-healthcare-for-india-100-a-century-of-change-on-the-horizon (Accessed June 27, 2024).

[B42] Taneja N. Sharma M. (2019). Antimicrobial resistance in the environment: the Indian scenario. Indian J. Med. Res. 49, 119–128. 10.4103/ijmr.IJMR_331_18 31219076 PMC6563737

[B43] Tello A. Austin B. Telfer T. C. (2012). Selective pressure of antibiotic pollution on bacteria of importance to public health. Environ. Health Perspect. 120, 1100–1106. 10.1289/ehp.1104650 22571927 PMC3440082

[B44] TNN (2002). Formulations and bulk drugs: get the basics right. The Economic Times. Available online at: https://economictimes.indiatimes.com/formulations-and-bulk-drugs-get-the-basics-right/articleshow/32797412.cms (Accessed April 22, 2024).

[B45] Uluseker C. Kaster K. M. Thorsen K. Basiry D. Shobana S. Jain M.. (2021). Review on occurrence and spread of antibiotic resistance in wastewaters and in wastewater treatment plants: mechanisms and perspectives. Front. Microbiol. 12:717809. 10.3389/fmicb.2021.717809 34707579 PMC8542863

[B46] United Nations (2024). The 17 Sustainable Development. United Nations. Available online at: https://sdgs.un.org/goals (Accessed July 12, 2024).

[B47] Walia K. Ohri V. C. Madhumathi J. Ramasubramanian V. (2019). Policy document on antimicrobial stewardship practices in India. Indian J. Med. Res. 149, 180–184. 10.4103/ijmr.IJMR_147_18 31219081 PMC6563731

[B48] World Health Organization (2019). No Time to Wait: Securing the Future from Drug-Resistant Infections: Report to the Secretary-General of the United Nations. Available online at: https://www.who.int/publications/i/item/no-time-to-wait-securing-the-future-from-drug-resistant-infections (Accessed August 1, 2025).

[B49] World Health Organization (2024). New Global Guidance Aims to Curb Antibiotic Pollution from Manufacturing. Available online at: https://www.who.int/news/item/03-09-2024-new-global-guidance-aims-to-curb-antibiotic-pollution-from-manufacturing (Accessed August 4, 2025).

[B50] Wu-Wu J. W. F. Guadamuz-Mayorga C. Oviedo-Cerdas D. Zamora W. J. (2023). Antibiotic resistance and food safety: perspectives on new technologies and molecules for microbial control in the food industry. Antibiotics 12:550. 10.3390/antibiotics12030550 36978417 PMC10044663

[B51] Yang Q. Gao Y. Ke J. Show P. L. Ge Y. Liu Y.. (2021). Antibiotics: an overview on the environmental occurrence, toxicity, degradation, and removal methods. Bioengineered 12, 7376–7416. 10.1080/21655979.2021.1974657 34612807 PMC8806427

[B52] Yang X. Zhong Q. Liang S. Li Y. Wang Y. Zhu X.. (2022). Global supply chain drivers of agricultural antibiotic emissions in China. Environ. Sci. Technol. 56, 5860–5873. 10.1021/acs.est.1c07110 35442028

[B53] Zhang M. (2023). In shortage: understanding global antibiotic supply chains through pharmaceutical trade fairs. Anthropologica 65, 1–16. 10.18357/anthropologica65120232605

[B54] Zhang R. Yang S. An Y. Wang Y. Lei Y. Song L.. (2022). Antibiotics and antibiotic resistance genes in landfills: a review. Sci. Total Environ. 806:150647. 10.1016/j.scitotenv.2021.150647 34597560

