# Supplementary material for: Mapping antibiotic pollution and tracking drivers of environmental AMR in a North Indian pharmaceutical hub
Source: Front Microbiol. 2025 Sep 15;16:1658029. doi: 10.3389/fmicb.2025.1658029 (PMC12477125; doi:10.3389/fmicb.2025.1658029)
Supplement: Supplementary file 1 [file Data_Sheet_1.PDF]

Title of Study: Resolving the fate and studying the impact of pharmaceutical wastes on the environment and local community of a pharmaceutical manufacturing hub.

Researcher: Dr. Amishi Panwar (amishi.panwar@bristol.ac.uk)

A. General - Health and Environment (in Baddi)

1. What are the most common health problems in the local population in your opinion?

2. Are there any health issues that you consider to be related to the local environment?

3. Are there any health issues that you consider to be related to people's living conditions?

B. Antimicrobial Use (AMU), Effectiveness and Disposal

4. Where do people generally go to seek care or treatment when ill?

5. General views about use of AntiMicrobials (AMs):

   Frequency & types of use

   Wide or limited availability ?

   Costs involved or free ?

6. General sense of the effectiveness of AMs in alleviating or curing symptoms.

7. General idea of method of disposing unused/expired medicines.

8. Use of AMs in animal feed?

   Types

   Usage

   Infections

C. Water, Sanitation & Sewage

9. Organisations and/or regulatory measures associated with environmental or pollution control.

10. Method of waste disposal in pharmaceutical factories:

waste treated and disposed according to legal safety norms ?

informal dumping in open areas ?

waste burned ?

liquid waste disposed through pipes that run off into open areas or underground ?

D. Effects of AM, APIs (Active Pharmaceutical Ingredients) and Waste (pharma) on the Environment & Humans

11. Perception of pharma waste in the community, any associated concerns, or problems.

Are these factories thought to affect/change the environment or human health?

Any known effects of pharma waste on the environment and human health? If so, describe in what ways?

What concerns are associated with these effects, if any?

12. Overall sources of pharmaceutical waste. Anything known about their effects on the natural and built environment ?

13. Anything known about their effects on human health? Any localised diseases or epidemics that can be traced to particular factories, workplaces or residential neighbourhoods:

related to skin, eye, breathing, digestive, bowel disorders;

individual cases, localised, epidemic, or endemic?

14. Any known effects of waste upon agriculture, livestock, pets, wild animals?

15. Whether pharma waste or APIs enter the environment and/or cause changes:

Known changes detected in soil, water, lakes, rivers, streams, forests;

understanding of the connections between disposal sites, water bodies (*bawris*, wells, water tanks) and consumption?

16. Whether plant & animal products consumed might be at risk of exposure to pharma waste or APIs?

concerns or complaints about the quality of these products;

proximity of livestock and/or agricultural land and markets and *chowks* to sources of APIs;

types and locations of sites.

E. Social, Political and Economic Drivers of Pharmaceutical Waste Management

17. Perception and understanding of the benefits of the pharmaceutical industry in area.

no perceived benefits

jobs created/ economic benefits

advancement of science

industrial growth

18. Response of local communities to the presence of the pharma industry.

General understanding that the industry is beneficial to the community

concern, dissatisfaction or even contestation

any advocacy to contest prevalent practices.

19. Laws and regulations regarding industry waste disposal –

existing laws e.g. the Common Effluent Treatment Plant (CETP)

gaps in laws

gaps between laws and implementation and issues neglected by existing legislation.

20. Economic pressures and/or dominance of pharma companies:

Jobs for community members;

bonuses for factory managers;

incentives offered to politicians and bureaucrats, if any?

21. Political pressures –

any influence of factory owners on the communities;

any intimidation of local communities?
